# In Vitro Mechanical Evaluation of Prosthodontic Dental Acrylics Fabricated by Conventional and Additive Techniques

**DOI:** 10.3390/ma19071413

**Published:** 2026-04-01

**Authors:** Paweł Szymlet, Wojciech Frąckiewicz, Konrad Kwiatkowski, Marcin Królikowski, Mirona Palczewska-Komsa, Małgorzata Kozak, Alicja Sobiraj-Daba’n, Ewa Sobolewska

**Affiliations:** 1 Apolonia Dental, 70-772 Szczecin, Poland; 2 Maxillo-Facial Surgery Clinic, Pomeranian Medical University in Szczecin, 71-252 Szczecin, Poland; 3 Department of Mechanics and Fundamentals of Machine Design, Faculty of Mechanical Engineering and Mechatronics, West Pomeranian University of Technology in Szczecin, 70-310 Szczecin, Poland; 4 Department of Manufacturing Engineering, Faculty of Mechanical Engineering and Mechatronics, West Pomeranian University of Technology in Szczecin, 70-310 Szczecin, Poland; 5 Department of Dental Prosthetics, Faculty of Medicine and Dentistry, Pomeranian Medical University in Szczecin, 70-111 Szczecin, Poland

**Keywords:** prosthesis, 3D printing, acrylic, hardness, flexural strength, tensile strength, density, impact strength

## Abstract

**Introduction:** Acrylic materials have been a staple in dental prosthetics for decades. Developments in manufacturing technology, including additive techniques, have led to the introduction of new types of acrylics, whose mechanical properties require detailed evaluation and comparison with conventional materials. **Aim of the study:** The aim of the study was to comparatively evaluate selected mechanical properties, such as hardness, impact strength, density, flexural strength, and tensile strength, of three types of acrylic materials used in prosthetics: 3D-printed acrylic, heat-cured acrylic, and cold-cured acrylic, in vitro. **Materials and Methods:** Three materials were used in the study: 3D-printed acrylic (NextDent Denture 3D+), heat-cured acrylic (made using the cupping method (Villacryl H Plus)), and cold-cured acrylic (made using the pouring method (Villacryl SP)). Ten samples (*n* = 30) were prepared from each material. Flexural strength, tensile strength, Young’s modulus, density, Vickers hardness, and impact strength were tested. **Results:** The tested materials demonstrated significant differences in mechanical properties. The highest values for impact strength, flexural strength, tensile strength, and density were obtained for 3D-printed acrylic. Hot-cured acrylic achieved the highest hardness values. Cold-cured acrylic, on the other hand, achieved the lowest results, except for the impact strength test, where it achieved intermediate results. **Conclusions:** The mechanical properties of dental acrylics are significantly dependent on their manufacturing technology. Hot-cured acrylic exhibits the highest hardness, while 3D-printed acrylic is characterized by favorable impact strength, flexural strength, and tensile strength, which may have significant clinical significance in terms of the resistance of dentures to mechanical damage. The study results could provide a basis for introducing this material into clinical practice.

## 1. Introduction

Polymethyl methacrylate (PMMA), commonly referred to as acrylic, is a synthetic polymer extensively employed in dental prosthodontics. Its origins can be traced to the nineteenth century, with the isolation of acrylic acid in 1843 and the subsequent synthesis of methacrylic acid in 1865. Large-scale industrial production commenced in 1928, marking a pivotal moment in the advancement of dental materials [1]. By the 1930s, PMMA had already been introduced into denture fabrication, owing to its favorable mechanical performance and aesthetic qualities [2]. Over subsequent decades, acrylic materials became the standard in prosthetic dentistry, supplanting earlier materials that were inferior in strength and appearance.

Acrylic materials have remained the primary material used in dental prosthetics for several decades. Heat-polymerized acrylics are regarded as the gold standard due to their favorable mechanical properties, low water absorption, and good stability under oral conditions [3]. In dentistry, acrylic has been used in the fabrication of dentures, crowns, bridges, and other prosthetic appliances [4]. Its properties, including ease of processing, aesthetic appearance, colorability, and biocompatibility, make it the material of first choice in many clinical cases [5].

However, despite its numerous advantages, acrylic also has certain limitations. It is susceptible to cracking and wear and exhibits limited resistance to chemical and thermal factors, which may affect the durability of dental prostheses [6]. Consequently, research is being conducted on the modification of acrylic materials, including reinforcement with glass fibers, the incorporation of nanoparticles, and alterations in the chemical composition of the resin, with the aim of improving the material’s strength and resistance [7].

There are various types of acrylic materials. Among the most commonly used in dental prosthetics for denture fabrication are heat-polymerizing and cold-polymerizing acrylics [8]. Heat-polymerizing acrylic has been traditionally employed in dentistry [9]. It is characterized by high mechanical strength, resistance to deformation, and good color stability; however, the polymerization process requires high temperatures, which may lead to microcracks and material distortions [10]. In contrast, cold-polymerizing (self-curing) acrylic is easier to manipulate, requires lower temperatures, and has a shorter processing time, but its mechanical properties, including flexural strength and hardness, are often inferior to those of heat-polymerizing acrylic [11].

Additive manufacturing approaches have recently become increasingly prevalent, facilitating accurate replication of anatomical details, shorter production times for dentures, and the fabrication of prototypes and customized patient-specific components [12]. 3D printing further allows for material optimization, reduced material usage, and enhanced consistency in prosthetic outputs [13]. Nonetheless, 3D printing resins still present some drawbacks; they typically possess lower flexural strength and hardness than heat-cured acrylics, although they frequently exhibit greater impact resistance, which may be beneficial in denture applications [14].

The mechanical properties of 3D-printed materials are closely dependent not only on their chemical composition but also on the parameters of the technological process. Factors such as print orientation, layer thickness, and exposure conditions have been shown to significantly influence the mechanical properties of the resulting structures. Research indicates that both printing parameters and post-processing procedures, including post-curing, play a key role in shaping the properties of materials used for denture bases. Changes in these parameters can lead to significant differences in the mechanical strength and surface properties of the materials [15].

Other studies analyzing additive manufacturing processes highlight that the properties of materials produced using additive manufacturing methods are strongly dependent on the process parameters and the resulting microstructure, which is often anisotropic in nature. As a consequence, these materials may exhibit different mechanical properties depending on the loading direction [16].

Acrylic printing techniques encompass stereolithography (SLA), digital light processing (DLP), and selective laser sintering (SLS) [17]. Each approach presents specific strengths and weaknesses: SLA and DLP provide high accuracy and smooth surface quality, while SLS facilitates the use of acrylic powders, enhancing the mechanical strength of printed parts, although it necessitates additional post-processing [18]. The selection of a suitable printing method is determined by clinical needs, material characteristics, and manufacturing expenses [19].

The mechanical characteristics of denture base materials—including flexural and tensile strength, hardness, impact resistance, and density—play a pivotal role in ensuring durability, safety during use, and patient comfort. Flexural strength is considered a key parameter, as removable prostheses experience repeated masticatory loading that may cause fatigue-related fractures [20,21]. Tensile strength represents the material’s capacity to resist stretching forces without initiating cracks, a factor of particular relevance in areas where the denture exhibits thin cross-sections [22].

The hardness of a denture base material has a direct influence on its resistance to wear and the long-term integrity of its surface, which is significant not only mechanically but also biologically, given the risk of biofilm and deposit retention [23,24]. Material density can serve as an indirect indicator of structural uniformity and the presence of internal voids, which may act as initiation points for cracks and compromise mechanical strength [25]. Impact resistance is a clinically critical property, representing the material’s capacity to absorb energy during sudden dynamic loads, such as accidental denture drops onto hard surfaces—one of the leading causes of prosthesis failure in clinical settings [26,27].

The aim of this article is to compare the mechanical properties of three types of acrylic: 3D-printed acrylic, cold-polymerizing acrylic, and heat-polymerizing acrylic [28]. Parameters such as hardness, impact resistance, flexural strength, and tensile strength will be analyzed to assess their suitability for dental applications and to identify potential directions for the development of prosthetic materials [29].

## 2. Materials and Methods

Three materials were compared for the study:

(1) 3D-printed acrylic—Nextdent Denture 3D+ Light Pink (NextDent, Vertex-Dental B.V., Soesterberg, The Netherlands)—**group 1**;
(2) Heat-polymerized acrylic using the flask method—Villacryl H Plus (Everall7, Warsaw, Poland)—**group 2**;
(3) Cold-polymerized acrylic using the pouring method into a silicone mold—Villacryl SP (Everall7, Warsaw, Poland)—**group 3**.

**Figure 1:**
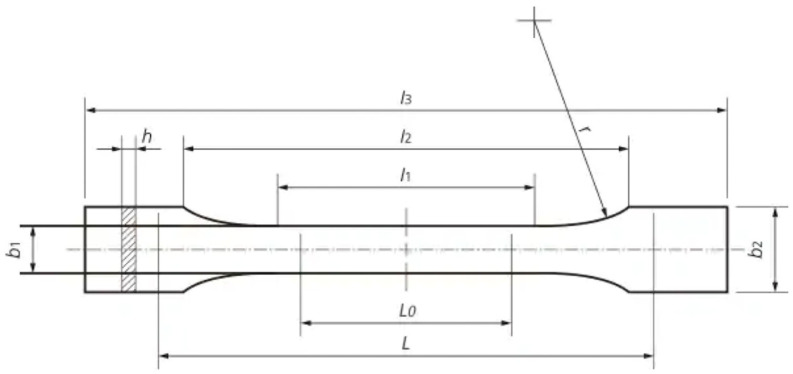
Shape and dimensions of the sample.

**Figure 2:**
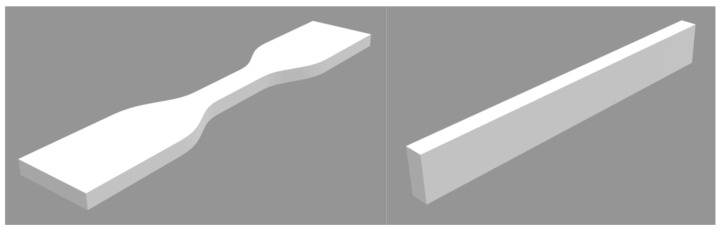
Schematic diagram of the samples produced, presented using an STL file.

Ten paddle-shaped specimens (Figure 1) and ten bar-shaped specimens (80 × 10 × 4 mm) were fabricated from each of these materials. The specimens were designed using dedicated software (Exocad Rijeka 3.1, Exocad GmbH, Darmstadt, Germany), and their specimen shape models (Figure 2) were exported in STL format.

The samples intended for material characterization had the shape of universal shapes (paddles) for testing polymeric materials in accordance with the PN-ISO 37:2005, type 3 standard [30]. The dimensions of these samples are presented in Table 1. The modification of the samples in this study involved extending the gripping part in order to mount them in the jaws of the testing machine.

For the preparation of specimens made of heat-polymerized conventional acrylic resin, the material Villacryl H Plus (Everall7, Warsaw, Poland) was used. It consists of a powder containing benzoyl peroxide and a liquid containing methyl methacrylate, tetramethylene dimethacrylate, and ethyl methacrylate. The specimens were fabricated using the conventional flasking technique. First, silicone molds of appropriate dimensions were prepared, in which wax patterns of the future specimens were formed. The wax specimens were placed in the lower part of the flask filled with dental stone, covered with an isolating layer to prevent adhesion between stone surfaces, and then closed with the upper part of the flask, also filled with stone. The flask was placed in boiling water to eliminate the wax. After opening and cooling the flask, the prepared and insulated stone mold was filled with acrylic dough mixed according to the manufacturer’s recommendations (24 g of powder to 10 g of liquid), prepared within 20–25 min at 23 °C. The flask was then closed, compressed using a hydraulic press, and polymerized according to the manufacturer’s recommended cycle: heating from 60 °C to 100 °C over 30 min, maintaining at 100 °C for 30 min, and finally cooling in air for 30 min. The finished specimens were deflasked and manually finished by the same operator. First, excess acrylic was removed, and edges were shaped using a bur, followed by wet grinding with abrasive papers of P500, P1000, and P1200 grit to achieve precise dimensions compliant with ISO standards, verified using an electronic caliper. These procedures were repeated until the required number of specimens was obtained.

For the preparation of specimens made of cold-polymerized acrylic resin, Villacryl SP (Everall7, Warsaw, Poland) was used. Similarly to the conventional resin, it consists of a powder containing benzoyl peroxide and a liquid containing methyl methacrylate, ethylene dimethacrylate, and N,N-dimethyl-p-toluidine. The specimens were fabricated using the pouring (casting) technique. Silicone molds of appropriate dimensions were also prepared. The material was then mixed according to the manufacturer’s recommendations (10 g of powder to 5 g of liquid). The mixture was poured into the silicone molds within 30–60 s at 23 °C. After filling, the molds were placed in a pressure polymerization unit and polymerized under the manufacturer’s recommended conditions: 30 min at 60 °C under a pressure of 2 bar, followed by gradual cooling. After preliminary finishing (removal of excess material), the specimens were conditioned in water at 50 °C for 24 h to reduce residual monomer content. After drying, the specimens were finished to obtain precise dimensions in the same manner as those fabricated using the flasking technique. The specimens were fabricated in a dental laboratory, while the final finishing was performed by the same operator as in the case of conventionally fabricated specimens.

The 3D-printed samples were produced using a Phrozen Sonic Mini 8K 3D printer (Phrozen Technology, Hsinchu, Taiwan) operating in SLA technology. The printing process involves layer-by-layer photopolymerization of light-curing resin using an LED light source and image masking via an LCD screen. The models were prepared in STL format and processed using compatible slicer software (CHITUBOX version 1.9.0, CBD Technology, Shenzhen, China). After printing, the samples underwent standard post-processing, including rinsing in isopropyl alcohol (IPA) to remove uncured resin, drying at room temperature, final polymerization in a UV chamber, and mechanical removal of the supports with surface smoothing. Final finishing was performed by the same operator and in the same manner as for the hot and cold samples.

Mechanical tests were performed on the samples, including:

- bending strength (Young’s modulus calculated),
- tensile strength (Young’s modulus calculated),
- density,
- hardness,
- impact strength.

### 2.1. Evaluation of Flexural Strength

**Figure 3:**
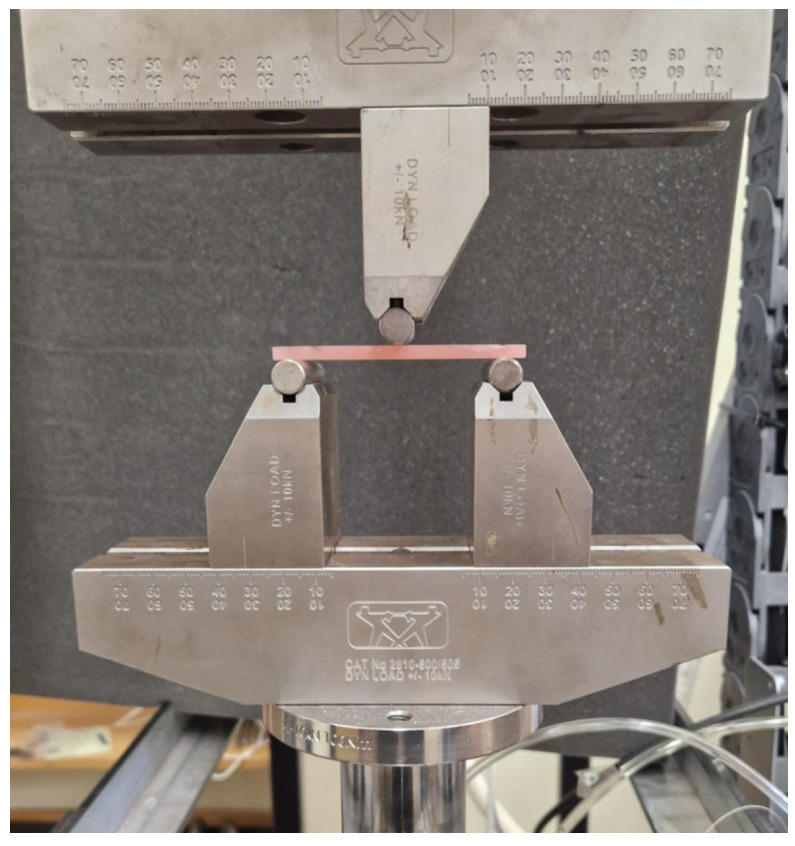
System for measuring flexural strength.

A three-point bending test was performed on a system consisting of two cylindrical supports placed at a distance of L = 64 mm. Five bar-shaped specimens from each group (*n* = 15) were used for the test, and measurements were taken using a universal testing machine (ElectroPlus E10000, Instron, Norwood, MA, USA). The cylindrical load cell was applied at the center of the specimen (Figure 3), and the loading rate was 5 mm/min. During the test, the maximum stress at which fracture occurred for each specimen was recorded, as well as the modulus of elasticity (Young’s modulus) for both materials.

### 2.2. Tensile Strength Assessment

**Figure 4:**
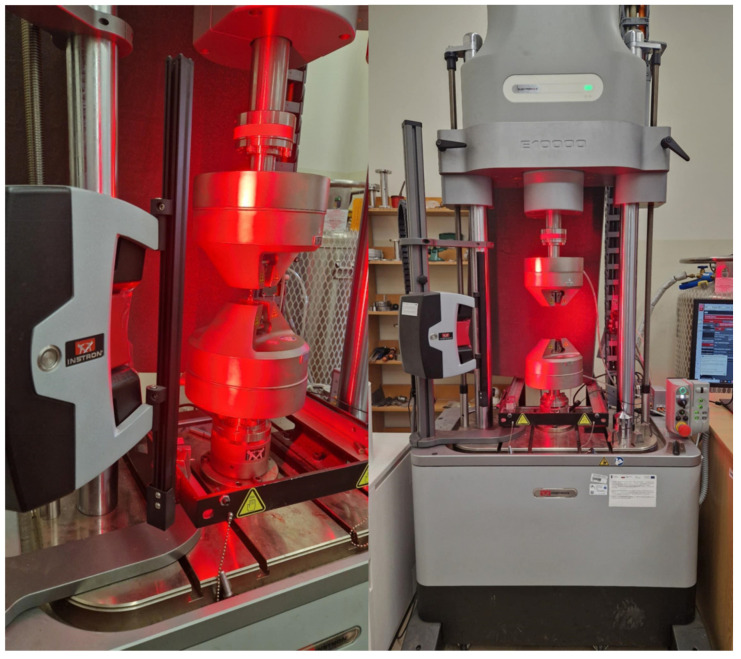
Tensile strength measurement system.

The tensile strength of acrylics from each group was measured. Five dumbbell-shaped samples from each group (*n* = 15) were used for testing. Measurements were taken using a universal tensile testing machine (ElectroPlus E10000, Instron, USA). The samples were tensile-stressed to fracture, and dimensional changes were monitored using a video extensometer. To facilitate camera visibility, the dumbbells were painted with a black marker and marked with two white dots on either side of the narrow section of the samples. Young’s modulus in compression was also calculated for the three material groups. The measurement was performed using the setup shown in Figure 4.

### 2.3. Density Assessment

**Figure 5:**
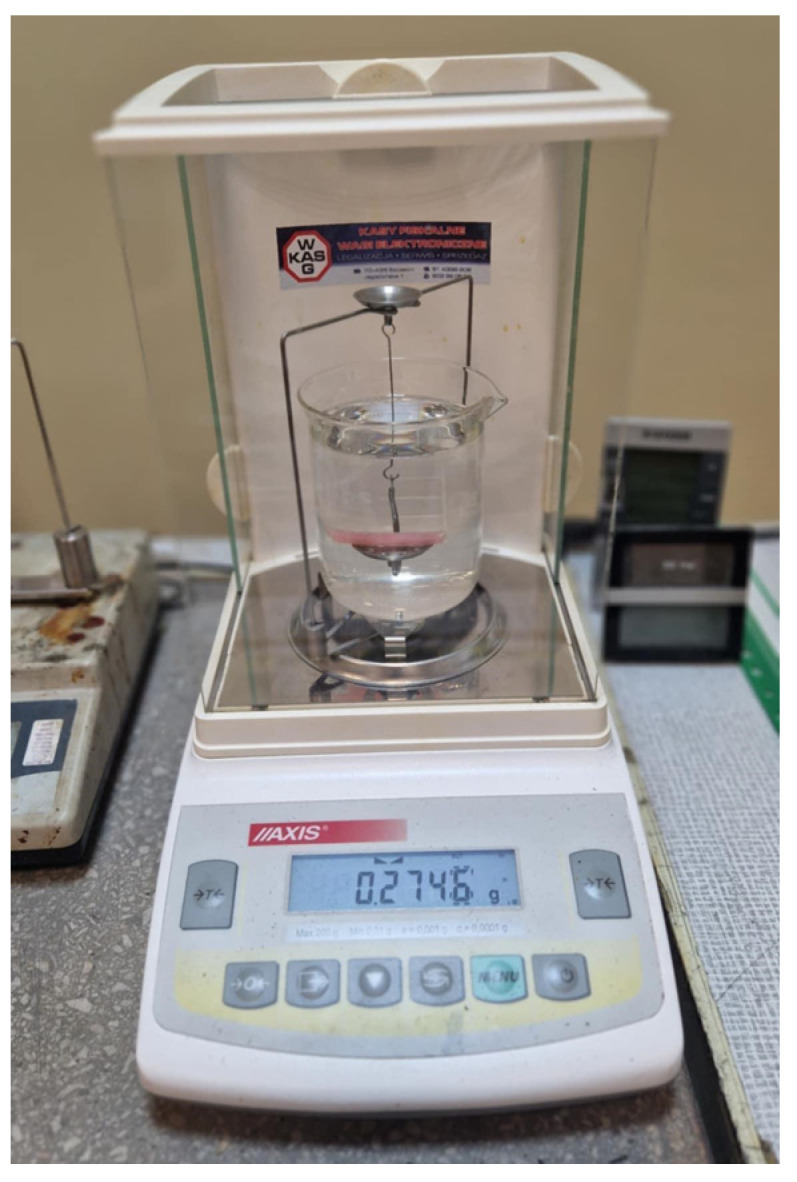
Density measurement system.

Density was measured using a laboratory hydrostatic balance (ALZ60, AXIS, Gdańsk, Poland), which includes an appropriate set for determining density with an accuracy of 0.015 g/cm^3^ (Figure 5), in accordance with the ISO 1183-1 standard [31]. Ten samples from each group (*n* = 30) were used for the measurements, which were leftovers from the impact test. The measurements were taken at 23 °C using the following formula:

$$d=\frac{m_{p}}{\left(m_{p}-m_{w\;}\right)}\cdot \;d_{w}+{\;d}_{p}$$

where m_p_ is the mass of the sample in air, m_w_ is the mass of the sample in water, d_w_ is the density of water at 23 °C (0.99766 g/cm^3^), and d_p_ is the density of air.

### 2.4. Hardness Assessment

**Figure 6:**
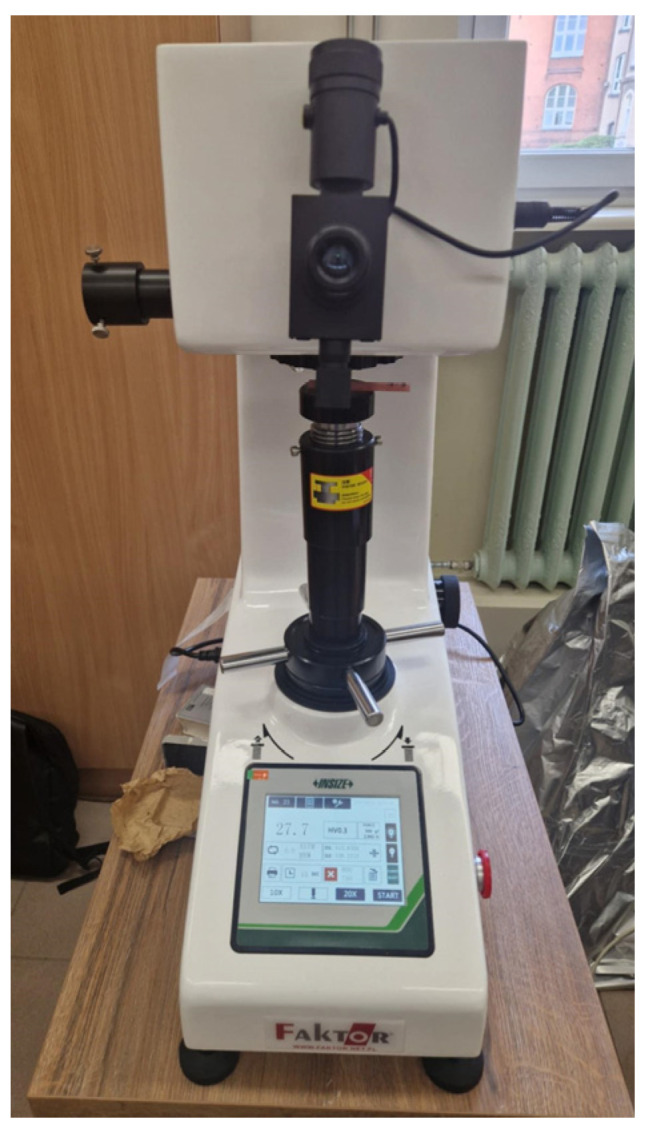
Hardness measuring system.

After the flexural strength test, the remaining bars were subjected to hardness measurements using a Digital Vickers Hardness Tester 8–3000 HV, HDT-VH51 (Insize Basic, Suzhou, China). Ten samples of each material (*n* = 30) were used for the test. After placing the sample on the test table, a cross-shaped cut was made using a metal indenter at a pressure of 300 g for 15 s. The distances between the cut ends were then measured using a microscope, and the computer-calculated values were displayed. Before and after the measurements, tests were performed on a 393 HV calibration plate. The measurement was performed using the setup shown in Figure 6.

### 2.5. Impact Strength Assessment

**Figure 7:**
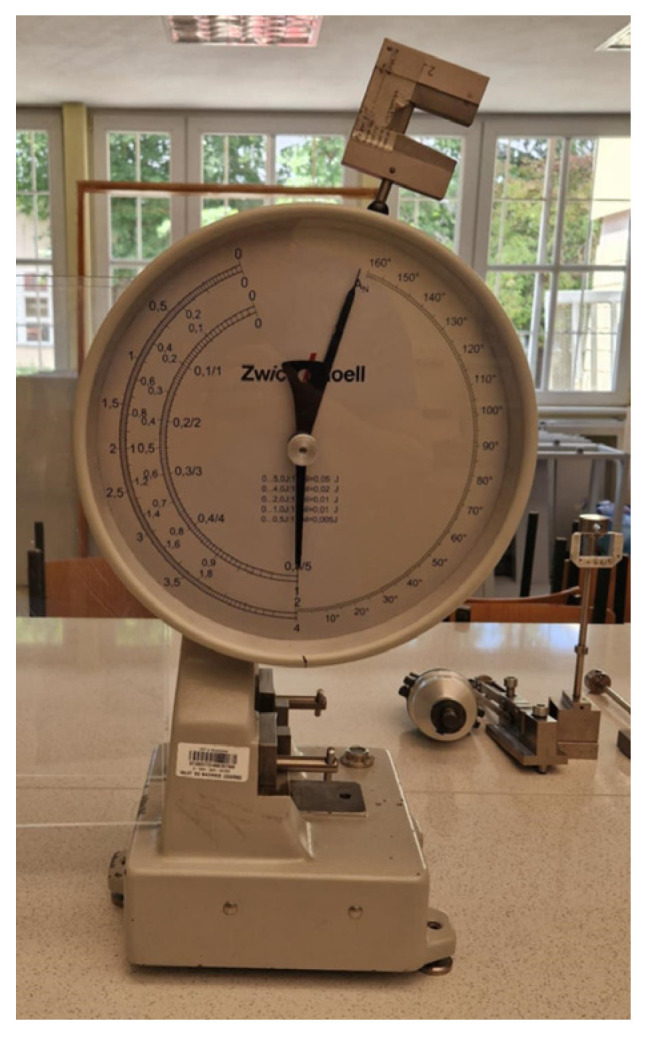
Impact strength measurement system.

A Fiat ZwickRoell impact tester was used to evaluate impact strength. The measurement was performed using the setup shown in Figure 7. Charpy impact strength: 2 J pendulum, 62 mm spacing. After placing the sample (without notch) on the supports in the device and releasing the hammer, the impact was applied, breaking the sample and rising to the other side. The results of the sample’s fracture work were read from analog indicators located on the device. The measurement was performed in accordance with ISO179-1 standards [32].

Impact strength was calculated using the formula:

$$K=\frac{W\;\cdot \;1000}{b\;\cdot \;h\;}$$

where

- W—work at fracture of the sample [J],
- b, h—width and thickness of the sample [mm].

### 2.6. Statistical Analysis

For descriptive analysis, the mean and standard deviation of a given acrylic group were calculated. Student’s *t*-test was used to assess the normality of data distribution (*p* < 0.01). The data were normally distributed. The comparison of the means of the results of both materials was performed based on the ISO 2854 standard [33], and the results for each characteristic are presented in table form in the Results Section, with a confidence interval at the 99% level.

## 3. Results

### 3.1. Evaluation of Flexural Strength

Mean flexural strength and standard deviation:

- Group 1 (3D): 107 ± 5.69 MPa;
- Group 2 (hot-cured): 96.1 ± 6.94 MPa;
- Group 3 (cold-cured): 78.2 ± 6.03 MPa.

The 3D-printed material exhibits the highest flexural strength, followed by the hot-cured material, and finally the cold-cured material. In practice, this means that when choosing a material that will withstand higher stresses and be more resistant to cracking during use (e.g., a prosthesis or structural component), it is better to choose a 3D-printed or hot-cured material over a cold-cured one (Table 2).

### 3.2. Tensile Strength Assessment

Mean tensile strength and standard deviation:

- Group 1 (3D): 68.0 ± 0.43 MPa;
- Group 2 (hot-cured): 64.1 ± 4.50 MPa;
- Group 3 (cold-cured): 47.6 ± 0.41 MPa.

The 3D-printed material exhibits the highest tensile strength, followed by the hot-cured material, and finally the cold-cured material. The 3D-printed material is the strongest and most resistant to tensile stress. This is important in applications where the material must withstand high stresses without fracture or deformation. The hot-cured material is also quite strong, but its properties can vary more between samples. The cold-cured material has the weakest tensile strength, so it may be less suitable for applications requiring high strength (Table 3).

### 3.3. Density Assessment

Average density:

- Group 1 (3D): 1.280 g/cm^3^;
- Group 2 (hot-cured): 1.185 g/cm^3^;
- Group 3 (cold-cured): 1.177 g/cm^3^.

3D-printed material has the highest density, followed by hot-cured polymerization, and finally cold-cured polymerization.

3D-printed material is the most compact, which may translate into greater mechanical resistance and lower water absorption. Hot-cured material is slightly less dense but still retains good mechanical properties. Cold-cured material is the most porous, which may result in lower strength and greater susceptibility to water absorption (Table 4).

### 3.4. Hardness Assessment

Mean hardness and standard deviation:

- Group 1 (3D): 21.39 ± 0.68 VHN;
- Group 2 (hot-cured): 25.39 ± 1.81 VHN;
- Group 3 (cold-cured): 17.66 ± 1.28 VHN.

The hardest material is hot-cured acrylic, followed by 3D-printed material, with cold-cured acrylic having the lowest values.

Hot-cured acrylic is the most scratch- and wear-resistant, making it the best choice for applications requiring high mechanical durability. 3D-printed material has medium hardness, which can be a compromise between mechanical resistance and ease of fabrication. Cold-cured acrylic is the least hard, so it will be more susceptible to mechanical damage, but its processing and preparation are typically simpler and faster (Table 5).

### 3.5. Impact Strength Assessment

Mean impact strength and standard deviation:

- Group 1 (3D): 11.4 ± 2.98 kJ/m^2^;
- Group 2 (hot): 8.7 ± 0.39 kJ/m^2^;
- Group 3 (cold): 9.2 ± 1.49 kJ/m^2^.

3D-printed material exhibits the highest impact strength, followed by cold-cured material, and hot-cured material has the lowest. For applications where impact resistance is important, the 3D material will be preferable. If repeatability and stability of properties are important, the hot-cured material will be more predictable due to smaller deviations from the mean. Cold-cured material, on the other hand, is a compromise between these two materials (Table 6).

**Table 3:** Tensile strength results.

Sample Number	Group 1 (3D)				Group 2 (Hot-Cured)				Group 3 (Cold-Cured)			
	Tensile Energy [J]	Tensile Stress at Maximum Force [MPa]	Relative Elongation at Maximum Stress [%]	Module (Segment 0.05–0.25%) [GPa]	Tensile Energy [J]	Tensile Stress at Maximum Force [MPa]	Relative Elongation at Maximum Stress [%]	Module (Segment 0.05–0.25%) [GPa]	Tensile Energy [J]	Tensile Stress at Maximum Force [MPa]	Relative Elongation at Maximum Stress [%]	Module (Segment 0.05–0.25%) [GPa]
1.	0.117	68.7	3.19	3.64	0.128	66.8	3.65	3.12	0.0705	48.1	3.85	2.14
2.	0.126	67.9	3.13	3.72	0.0811	59.0	2.67	3.42	0.0606	47.7	3.83	2.33
3.	0.162	67.8	3.35	3.56	0.108	66.5	3.46	3.24	0.0717	47.6	3.98	2.29
4.	0.186	67.5	3.64	3.58	0.131	68.8	4.08	3.16	0.0907	47.0	3.84	2.49
5.	0.142	68.2	3.00	3.55	0.0807	59.6	2.64	3.33	0.127	47.3	3.80	2.59
Mean	0.147	68.0	3.26	3.61	0.106	64.1	3.30	3.25	0.0841	47.6	3.86	2.37
Median	0.142	67.9	3.19	3.58	0.108	66.5	3.46	3.24	0.0717	47.6	3.84	2.33
Standard deviation	0.03	0.43	0.25	0.07	0.02	4.50	0.63	0.12	0.03	0.41	0.07	0.18
Coefficient of variation [%]	19.0	0.6	7.5	2.0	23.1	7.0	19.2	3.7	31.4	0.9	1.8	7.4

**Table 4:** Density results.

Sample Number	Group 1 (3D)			Group 2 (Hot-Cured)			Group 3 (Cold-Cured)		
	mp [g]	mw [g]	d [g/cm^3^]	mp [g]	mw [g]	d [g/cm^3^]	mp [g]	mw [g]	d [g/cm^3^]
1.	1.75	0.387	1.281	1.749	0.276	1.185	1.702	0.259	1.177
2.	1.629	0.358	1.279	1.773	0.281	1.186	1.393	0.212	1.177
3.	1.593	0.352	1.281	1.69	0.266	1.184	1.69	0.256	1.176
4.	1.896	0.418	1.280	1.538	0.243	1.185	1.669	0.254	1.177
5.	1.801	0.397	1.280	1.743	0.275	1.185	1.54	0.235	1.178
6.	1.796	0.395	1.279	1.726	0.271	1.184	1.766	0.269	1.177
7.	1.777	0.392	1.280	1.569	0.248	1.185	1.651	0.252	1.178
8.	1.667	0.367	1.280	1.747	0.276	1.185	1.54	0.234	1.177
9.	1.842	0.406	1.280	1.631	0.257	1.185	1.566	0.238	1.177
10.	1.964	0.433	1.280	1.604	0.253	1.185	1.512	0.23	1.177

**Table 5:** Hardness results.

Sample Number	Group 1 (3D)			Group 2 (Hot-Cured)			Group 3 (Cold-Cured)		
	Measurement 1	Measurement 2	Mean	Measurement 1	Measurement 2	Mean	Measurement 1	Measurement 2	Mean
1.	20.5	21.3	20.9	27.2	25.8	26.5	16.3	17	16.65
2.	21.4	23.1	22.25	28.1	27.7	27.9	17.8	18.4	18.1
3.	22.4	21.2	21.8	22.4	23.1	22.75	20.4	19.8	20.1
4.	20.2	21.8	21	26.5	23	24.75	19.1	17.8	18.45
5.	21.2	20	20.6	24.8	23.2	24	16.5	17.9	17.2
6.	21	20.4	20.7	26.3	24.7	25.5	18.1	20.2	19.15
7.	21.6	19.8	20.7	22.9	22.5	22.7	17.4	16.9	17.15
8.	21.3	23.1	22.2	27.1	26.1	26.6	18.4	16.2	17.3
9.	21.3	22.9	22.1	26.7	25	25.85	15.6	16.6	16.1
10.	22	21.3	21.65	25.7	29	27.35	15.7	17.1	16.4

**Table 6:** Impact strength results.

Sample Number	Group 1 (3D)				Group 2 (Hot-Cured)				Group 3 (Cold-Cured)			
	b	h	W	Impact Strength [kJ/m^2^]	b	h	W	Impact Strength [kJ/m^2^]	b	h	W	Impact Strength [kJ/m^2^]
1.	Width [mm]	Thickness [mm]	Work [J]		Width [mm]	Thickness [mm]	Work [J]		Width [mm]	Thickness [mm]	Work [J]	
2.	9.4	3.97	0.49	13.13039	8.83	4.33	0.34	8.892632	9.1	4.1	0.37	9.9
3.	9.39	3.97	0.53	14.21739	8.76	3.86	0.29	8.576431	9.27	4.11	0.29	7.6
4.	9.4	4.01	0.39	10.34647	8.75	4.22	0.34	9.207854	9.2	3.7	0.38	11.2
5.	9.38	3.96	0.29	7.807284	8.92	4.25	0.31	8.177262	9.37	3.89	0.29	8.0
Mean	9.41	3.99	0.29	7.723879	8.6	4.1	0.3	8.508225	9.36	4.07	0.32	8.4
Median				11.4				8.7				9.2
Standard deviation				2.98				0.39				1.49
Coefficient of variation [%]				26.2				4.5				16.3

### 3.6. Statistical Analysis

The comparison of mean values for individual mechanical properties was performed according to the ISO 2854 standard. The results are presented in Table 2, where 99% confidence intervals (CI) for the differences between groups were calculated (Table 7).

Most differences between material groups for tensile strength, density, and hardness are statistically significant (*p* < 0.01) according to ISO 2854 and 99% confidence intervals. However, for impact strength, although the means differ, due to the large variability of 3D material results, the differences do not always reach statistical significance.

## 4. Discussion

### 4.1. Flexural Strength

The results of this study showed that the highest flexural strength was achieved by materials produced by 3D printing, followed by hot-cured acrylics, while the lowest values were recorded for cold-cured materials. This pattern of results is consistent with some available in vitro studies, which demonstrate that appropriately selected 3D-printed materials, with optimal process parameters and proper post-curing, can achieve flexural strength and other mechanical parameters comparable to, and in some cases even higher than, materials produced by conventional methods. These results suggest that 3D printing represents a promising technological and material alternative in dental prosthetics, particularly in the context of further development and standardization of this technology in the field of dental prosthetics. These studies have demonstrated that modern 3D printing resins can achieve very high flexural strength values, especially with optimal print orientation and proper post-processing [9,25].

In some studies, including our own research, heat-cured acrylics achieved lower flexural strength values compared to 3D-printed materials [27,34]. At the same time, it should be emphasized that conventional heat-cured acrylics remain materials with well-documented, stable, and repeatable mechanical properties. Numerous studies have demonstrated that they meet standard requirements for denture bases, including appropriate flexural strength values, confirming their long-term clinical usefulness. Despite the dynamic development of 3D printing technology, conventional materials still represent a benchmark in the evaluation of new prosthetic solutions, particularly in terms of predictable mechanical behavior and service life [35,36,37].

Cold-cured acrylic exhibits a lower conversion rate than heat-cured material, resulting in the presence of residual monomer that is susceptible to leaching. It has a lower degree of polymerization, which negatively impacts its mechanical properties, such as flexural strength [38,39].

Recent studies have shown that additive manufacturing parameters, particularly the orientation of layers relative to the loading direction, have a significant impact on the physical and mechanical properties of 3D-printed denture bases. Systematic literature reviews indicate that horizontal orientation (0°) generally increases flexural strength, while inclined and vertical orientations can lead to decreased strength and greater variability, which is related to varying interlayer adhesion and material microstructure. Studies show that changing the printing angle can significantly modify flexural strength values and affect compliance with the minimum requirements of ISO standards for denture base materials [40].

Additionally, post-curing parameters (time and temperature) modulate material properties, including surface hardness and degree of polymerization, although this effect is usually smaller than the effect of layer orientation [41].

### 4.2. Tensile Strength

Assessing the tensile strength of acrylics used to fabricate denture bases is an important element of the mechanical characterization of these materials, even though bending and compressive forces dominate clinical loading. However, tensile strength testing provides crucial information regarding the material’s behavior under localized tensile stresses, which occur in full and partial dentures during chewing, insertion, and removal of restorations, and as a result of uneven support of the denture base [42,43].

Research indicates that cracks and fractures in denture bases often initiate in areas of tensile stress concentration, such as around clasps, alveolar ridges, or areas of variable material thickness. Therefore, tensile strength is an important parameter for assessing the material’s resistance to the initiation and propagation of microcracks, which can lead to damage to dentures [44].

Our own research has shown that cold-cured acrylics have lower tensile strength compared to hot-cured materials, which is attributed to a lower degree of monomer conversion, greater porosity, and the presence of residual monomer that weakens the polymer structure. At the same time, modern resins used in 3D printing, thanks to the controlled layered manufacturing process and a more uniform microstructure, may exhibit better resistance to tensile stresses [45], which was also observed in our own study.

Studies show that 3D-printed polymeric materials exhibit anisotropic behavior, which means that their mechanical properties depend on the direction of the layers relative to the tensile forces [46].

### 4.3. Density

Currently, there is still a lack of studies analyzing the density of acrylic materials, both conventional and digitally manufactured. The vast majority of publications focus on assessing mechanical strength, water sorption, solubility, and porosity, treating these parameters as the main indicators of material quality. However, a material’s density is closely linked to its microstructure, degree of porosity, and water absorption capacity, which have direct clinical significance. Increased porosity of acrylic materials promotes greater water sorption, reduced mechanical properties, and an increased risk of microorganism colonization on the prosthesis surface. These parameters influence the durability of prosthetic restorations, their dimensional stability, and patient comfort. Despite numerous reports on the impact of manufacturing technology on the microstructure and physical properties of denture base resins, direct comparisons of the density of 3D-printed materials and conventional acrylics are still scarce, indicating a significant research gap and justifying the need for further research in this area. In our own study, the highest density was observed in the group of 3D-printed materials, followed by hot-cured acrylics, and the lowest in cold-cured materials. This trend is increasingly observed in scientific studies on the microstructure and physical properties of resins used in denture bases [27].

Additively printed materials are manufactured by controlled curing of successive resin layers, which promotes a more uniform and less porous structure compared to hand-mixing and polymerization techniques, which are more difficult to control in terms of micropore formation and phase inhomogeneity [47].

Conventional heat-cured acrylics also exhibit a compact structure with fewer pores than chemically autopolymerizing materials, as demonstrated by the own research results. As studies by other authors show, this is due to a higher degree of monomer conversion and a lower amount of residual monomer [48,49,50].

Cold polymerized materials, despite meeting mechanical standards, are usually characterized by greater porosity and less uniformity in the microstructure, which translates into lower density, as evidenced by our own studies, as well as greater water sorption and the potential degradation of physical properties in the oral environment [51].

### 4.4. Hardness

In the conducted studies, the highest hardness values were obtained for conventional heat-cured acrylics, followed by additively printed materials, while the lowest values were observed in the group of chemically polymerized materials. This distribution of results has been repeatedly confirmed in studies by other authors on the mechanical properties of denture base materials. Comparative studies have shown that heat-cured acrylics are characterized by significantly higher surface hardness compared to 3D-printed resins and autopolymerizing materials, which is associated with a higher degree of monomer conversion and a more compact polymer network obtained through the thermal polymerization process [52,53,54].

3D-printed resins, despite increasingly improved mechanical parameters, typically exhibit lower hardness values than conventional acrylics, which is explained by the layered nature of production and potential differences in the degree of hardening between individual material layers [55,56]. At the same time, numerous studies indicate that additive materials achieve significantly higher hardness values than chemically polymerized acrylics, which, due to the lower degree of polymerization and higher residual monomer content, are characterized by worse surface properties [50,57]. The results obtained in this study therefore confirm that conventional heat-cured acrylics remain the materials with the most stable mechanical properties in terms of hardness, while 3D printing is a promising alternative, and cold-polymerized materials exhibit the least favorable parameters in this respect.

The impact on hardness results should be interpreted in the context of the polymerization of acrylic materials, which directly affects the structure of the polymer network. Studies have shown that the monomer side effects lead to the formation of a more compact and utilized structure, which translates into both hardness and material failure [58].

It has been shown that the residual monomer is a result of mechanical action, acting as a plasticizing agent, which reduces hardness and increases the material’s susceptibility to degradation in the environment [59].

An important reason is what happens when polymerization occurs (post-curing), which can extend the range of 3D-printed materials and lead to their applications, including hardness [41,60].

Furthermore, it has been shown that the quality of workmanship, including the polishing procedure, affects the functional properties of materials, such as the occurrence of damage and durable clinical protection, which is important for specific performance [47,61].

The hardness values obtained as a result of the bonding, which are analyzed, are consistent with literature reports and may result not only from the manufacturing technology but primarily from differences in polymerization height, residual monomer content, and the final processing procedures.

### 4.5. Impact Strength

Impact strength is one of the key mechanical properties of materials used for denture bases, as it directly reflects the material’s ability to absorb impact energy without brittle fracture. Clinically, this is crucial in the context of common causes of denture failure, such as accidental dropping, sudden occlusal overloads, or uneven transfer of chewing forces, particularly in elderly patients [62,63]. Numerous in vitro studies have shown that low impact strength of the base material is a significant factor predisposing to fractures in full and partial dentures, regardless of their proper clinical fit [64].

In our own study, the highest impact strength was demonstrated by the 3D-printed material, followed by chemically polymerized acrylic, while the lowest values were obtained for conventional heat-cured acrylic. This pattern of results is confirmed by studies by other authors, although it should be emphasized that published data on the impact strength of additively printed materials are inconclusive. Some authors point to the lower impact strength of 3D-printed resins compared to traditional PMMA acrylics, which is explained by the layered structure of the material and potential anisotropy of mechanical properties [65,66]. At the same time, a growing number of studies indicate that modern 3D printing resins can achieve impact strength comparable to or even higher than classic thermally polymerized acrylics, as emphasized by our own results [67].

The high impact strength of the 3D-printed material obtained in this study can be interpreted in the context of a more uniform microstructure and reduced porosity compared to canned materials. Microscopic studies by other authors have shown that heat-polymerized acrylics can contain micropores and micro-stresses formed during thermal cycling, which act as crack initiation sites under dynamic loading [68,69].

Chemically polymerized acrylics exhibited intermediate impact strength values in this study, consistent with many previous publications. These materials are typically characterized by a lower elastic modulus and greater susceptibility to plastic deformation than conventional acrylics, which facilitates impact energy dissipation [70]. At the same time, the literature clearly indicates that the higher residual monomer content and greater porosity of these materials may limit their long-term mechanical stability, despite relatively favorable impact parameters [71].

From a clinical perspective, the obtained results suggest that materials with higher impact strength, including modern 3D-printed materials, may potentially reduce the risk of prosthetic failure resulting from impact loading. However, it should be emphasized that impact strength is only one of the parameters determining the clinical usefulness of the base material and should be interpreted in conjunction with other mechanical, physicochemical, and biological properties [9,72].

### 4.6. Clinical Significance and Limitations of the Study

Our own results indicate that 3D-printed materials can be a valuable alternative to conventional acrylics for denture production, offering favorable mechanical properties. It should be emphasized that this study was conducted in vitro, and material properties may change under clinical conditions.

The results of this in vitro study provide important information on the mechanical properties of materials used as denture bases, but their clinical significance must be interpreted taking into account differences between laboratory conditions and actual loading in the oral cavity.

One limitation of this study is the lack of a priori statistical power analysis to determine the optimal sample size. This may impact the ability to detect subtle differences between the tested materials, particularly for parameters characterized by greater variability, such as impact strength.

All 3D-printed samples were produced with a uniform orientation relative to the build platform, with their main dimensions set so that the layers grew in a constant direction (i.e., horizontally relative to the printer base). Literature indicates that print orientation affects the mechanical properties of 3D-printed materials. Studies on denture bases have found that samples printed in a horizontal orientation (0°), parallel to the build platform, often exhibit higher flexural strength than those printed at other angles (e.g., 45° or 90°), which is attributed to differences in interlayer adhesion and the degree of bonding between successive layers of material [40].

However, it should be noted that the sample size used (*n* = 10) falls within the range of values commonly used in in vitro studies on prosthetic materials, allowing for comparison of results with literature data. Despite the lack of a power analysis, the differences observed for most of the analyzed parameters were statistically significant (*p* < 0.01), suggesting that the sample size was sufficient to detect main effects.

It should be emphasized that the use of unnotched specimens limits the ability to directly compare results with literature data using notched specimens. Tests using notched specimens typically yield lower impact strength values because the notch acts as a stress concentrator and crack initiator. According to ISO 179-1 [32], results obtained for different specimen geometries (notched and unnotched) are not directly comparable because the mechanism of crack initiation and propagation varies. Therefore, comparisons of the results of this study with studies using notched specimens should be interpreted with caution. Differences in test methodology may lead to different impact strength values, regardless of the actual material properties.

Another aspect is the reproducibility of sample production. Traditional heat-cured acrylic production techniques require the flask and hand-mixing of components, making it difficult to ensure identical, repeatable test samples. This can lead to variability in results due to differences in mixing technique, temperature control, and polymerization time, as well as the possibility of introducing air bubbles and pores in the material structure [69,73]. In contrast, 3D printing enables precise, automated production of samples with high geometric reproducibility and controlled microstructure, which can improve the reliability of mechanical comparisons in laboratory and clinical settings [74,75].

In this study, mechanical property measurements were performed exclusively on as-fabricated samples, without subjecting them to aging processes such as water sorption, hydrolysis, or temperature cycling. This choice stems from the fact that the aim of the study was to determine the initial properties of acrylic materials immediately after sample fabrication, before the oral environment, characterized by high humidity and variable temperature, begins to operate. Analysis of the influence of aging factors remains the subject of separate studies.

Despite the rapid development of additive technologies, limitations include the scarcity of extensively documented clinical studies on the long-term behavior of 3D materials in the oral cavity. Consequently, there is a need for further clinical studies and long-term observations on the behavior of 3D-printed dentures under real-world conditions, particularly in the context of time, wear, material aging, retention, and patient comfort. Such studies would be essential to assess their durability, risk of fracture, and need for repair compared to conventional dentures, which represents a significant gap for further research [76,77].

In future studies, it is recommended to conduct a statistical power analysis at the experimental planning stage and increase the sample size, which will allow for a more precise assessment of differences between materials.

Clinical studies with long-term follow-up have shown that 3D-printed materials undergo changes over time, including changes in color and surface properties after use in the oral cavity, indicating a significant impact of material aging on their clinical properties [78].

Studies have shown that aging reduces the mechanical properties of denture base materials, including hardness, flexural strength, and fracture toughness, which directly impacts their clinical durability and susceptibility to damage during use. These changes are crucial for assessing their long-term effectiveness in prosthetic treatment [79].

Patient-reported outcomes are also an important element in assessing treatment effectiveness. Studies have shown that overall patient satisfaction with 3D-printed dentures is comparable to conventional dentures, although in some respects, such as aesthetics and phonetics, traditional dentures may receive higher ratings [80].

Therefore, the interpretation of in vitro test results should consider not only the mechanical properties of the materials but also their behavior in clinical settings, including aging processes, changes in properties over time, and patient subjective experiences. This highlights the need for further long-term clinical studies to fully assess the durability and suitability of 3D-printed materials in dental prosthetics.

## 5. Conclusions

3D-printed material is characterized by the highest flexural and tensile strength, followed by heat curing, and finally cold curing. In practice, this means that when manufacturing prostheses or structural components, where resistance to higher stresses and cracks during use is expected, it is better to choose a 3D-printed or heat-cured material. 3D-printed material also exhibits the highest density and impact strength, which translates into greater mechanical strength and lower water absorption, which clinically can limit material degradation, reduce the risk of microbial colonization, and improve the hygiene and durability of the prosthesis. Heat-cured acrylic is the hardest and exhibits the smallest property fluctuations, indicating its stability and durability. Clinically, this indicates high dimensional stability and predictable long-term performance. Cold-cured material, despite being easier to process, exhibits the lowest strength and hardness, limiting its use in demanding conditions. Clinically, this means its use is limited to temporary, repair, or less stressed prosthetic components.

Statistical analysis confirmed that most differences in strength, density, and hardness between the materials were statistically significant (*p* < 0.01), confirming the reliability and significance of the observed differences in the context of material selection. From a clinical perspective, this is very important, as it justifies informed material selection based on anticipated loading conditions and the expected durability of the prosthetic restoration.

## Figures and Tables

**Table 1:** Sample dimensions.

Symbol	Explanation of the Symbol	Dimensions [mm]
l_3_	Total length	58 ± 0.5
l_2_	Distance between wide parallel parts	32 ± 0.5
l_1_	The length of the part bounded by parallel lines	19 ± 0.5
r	Radius	≥8
b_1_	Width of the narrow part	4 ± 0.5
b_2_	Width at the ends	10 ± 0.5
h	Thickness	2
L_0_	Measuring length	15 ± 0.5
L	The initial distance between the handles	38 ± 0.5

**Table 2:** Flexural strength results.

Sample Number	Group 1 (3D)					Group 2 (Hot-Cured)					Group 3 (Cold-Cured)				
	Displacement at Maximum Force [mm]	Bending Deformation (Displacement) at Maximum Force [%]	Young’s Modulus Bending Stress 0.05–0.25%) [GPa]	Maximum Strength [N]	Bending Stress at Maximum Force [MPa]	Displacement at Maximum Force [mm]	Bending Deformation (Displacement) at Maximum Force [%]	Young’s Modulus Bending Stress 0.05–0.25%) [GPa]	Maximum Strength [N]	Bending Stress at Maximum Force [MPa]	Displacement at Maximum Force [mm]	Bending Deformation (Displacement) at Maximum Force [%]	Young’s Modulus Bending Stress 0.05–0.25%) [GPa]	Maximum Strength [N]	Bending Stress at Maximum Force [MPa]
1	5.71	3.30	3.77	163	107	5.39	3.20	3.13	128	87.0	8.10	4.30	2.78	109	86.1
2	5.96	3.49	3.64	168	109	6.50	4.02	3.16	167	100	9.42	5.56	2.64	133	82.8
3	5.14	2.97	3.64	150	98.6	6.54	4.21	3.24	185	105	6.28	3.38	2.76	99.3	76.1
4	5.51	3.18	3.70	159	105	5.68	3.51	3.31	157	94.9	6.18	3.50	2.54	103	71.8
5	6.46	3.73	3.72	173	114	5.71	3.45	3.21	150	93.2	5.75	3.40	2.71	116	74.2
Mean	5.76	3.33	3.70	162	107	5.96	3.68	3.21	158	96.1	7.15	4.03	2.69	112	78.2
Median	5.71	3.30	3.70	163	107	5.71	3.51	3.21	157	94.9	6.28	3.50	2.71	109	76.1
Standard deviation	0.50	0.29	0.06	8.98	5.69	0.53	0.42	0.07	21.01	6.94	1.56	0.94	0.10	13.21	6.03
Coefficient of variation [%]	8.65	8.77	1.56	5.53	5.34	8.81	11.48	2.16	13.34	7.22	21.81	23.26	3.59	11.78	7.71

**Table 7:** Statistical analysis of results.

Flexural strength [MPa]			
Group	Mean (MPa)	SD	99% CI (mean)
3D	107	5.69	106.72 ± 12.52
Hot-cured	96.1	6.94	96.02 ± 14.27
Cold-cured	78.2	6.03	78.20 ± 12.43
Tensile strength [MPa]			
Group	Mean (MPa)	SD	99% CI (mean)
3D	68.0	0.43	67.7–68.3
Hot-cured	64.1	4.50	59.8–68.4
Cold-cured	47.6	0.41	47.3–47.9
Density [g/cm^3^]			
Group	Mean (g/cm^3^)		
3D	1.280		
Hot-cured	1.185		
Cold-cured	1.177		
Hardness [VHN]			
Group	Mean (VHN)	SD	99% CI (mean)
3D	21.39	0.68	20.1–22.7
Hot-cured	25.39	1.81	22.4–28.4
Cold-cured	17.66	1.28	15.0–20.3
Impact strength [kJ/m^2^]			
Group	Mean (kJ/m^2^)	SD	99% CI (mean)
3D	11.4	2.98	5.2–17.6
Hot-cured	8.7	0.39	7.8–9.6
Cold-cured	9.2	1.49	5.9–12.5

## Data Availability

The raw data supporting the conclusions of this article will be made available by the authors on request.

## Abbreviations

The following abbreviations are used in this manuscript:

3D	Three-Dimensional
CI	Confidence Interval
DLP	Digital Light Processing
ISO	International Organization for Standardization
MD	Mean Deviation
PMMA	Polymethyl Methacrylate
SD	Standard Deviation
SLA	Stereolithography
SLS	Selective Laser Sintering
STL	Stereolitography
